# Carbapenem-Nonsusceptible *Acinetobacter baumannii*, 8 US Metropolitan Areas, 2012–2015

**DOI:** 10.3201/eid2404.171461

**Published:** 2018-04

**Authors:** Sandra N. Bulens, Sarah H. Yi, Maroya S. Walters, Jesse T. Jacob, Chris Bower, Jessica Reno, Lucy Wilson, Elisabeth Vaeth, Wendy Bamberg, Sarah J. Janelle, Ruth Lynfield, Paula Snippes Vagnone, Kristin Shaw, Marion Kainer, Daniel Muleta, Jacqueline Mounsey, Ghinwa Dumyati, Cathleen Concannon, Zintars Beldavs, P. Maureen Cassidy, Erin C. Phipps, Nicole Kenslow, Emily B. Hancock, Alexander J. Kallen

**Affiliations:** Centers for Disease Control and Prevention, Atlanta, Georgia, USA (S.N. Bulens, S.H. Yi, M.S. Walters, A.J. Kallen);; Emory University School of Medicine, Atlanta (J.T. Jacob);; Georgia Emerging Infections Program, Atlanta (J.T. Jacob, C. Bower, J. Reno);; Atlanta Veterans Affairs Medical Center, Decatur, Georgia, USA (C. Bower, J. Reno); Atlanta Research and Education Foundation, Decatur (C. Bower, J. Reno);; Maryland Department of Health and Mental Hygiene, Baltimore, Maryland, USA (L. Wilson, E. Vaeth);; Colorado Department of Public Health and Environment, Denver, Colorado, USA (W. Bamberg, S.J. Janelle); Minnesota Department of Health, St. Paul, Minnesota, USA (R. Lynfield, P.S. Vagnone, K. Shaw);; Tennessee Department of Public Health, Nashville, Tennessee, USA (M. Kainer, D. Muleta, J. Mounsey);; New York Rochester Emerging Infections Program at the University of Rochester Medical Center, Rochester, New York, USA (G. Dumyati, C. Concannon);; Oregon Health Authority, Portland, Oregon, USA (Z. Beldavs, P.M. Cassidy);; University of New Mexico, Albuquerque, New Mexico, USA (E.C. Phipps, N. Kenslow, E.B. Hancock)

**Keywords:** antimicrobial resistance, carbapenems, carbapenem-nonsusceptible, surveillance, prevention, bacteria, Acinetobacter baumannii, Acinetobacter infections, United States, Emerging Infections Program

## Abstract

In healthcare settings, *Acinetobacter* spp. bacteria commonly demonstrate antimicrobial resistance, making them a major treatment challenge. Nearly half of *Acinetobacter* organisms from clinical cultures in the United States are nonsusceptible to carbapenem antimicrobial drugs. During 2012–2015, we conducted laboratory- and population-based surveillance in selected metropolitan areas in Colorado, Georgia, Maryland, Minnesota, New Mexico, New York, Oregon, and Tennessee to determine the incidence of carbapenem-nonsusceptible *A. baumannii* cultured from urine or normally sterile sites and to describe the demographic and clinical characteristics of patients and cases. We identified 621 cases in 537 patients; crude annual incidence was 1.2 cases/100,000 persons. Among 598 cases for which complete data were available, 528 (88.3%) occurred among patients with exposure to a healthcare facility during the preceding year; 506 (84.6%) patients had an indwelling device. Although incidence was lower than for other healthcare-associated pathogens, cases were associated with substantial illness and death.

The bacterium *Acinetobacter baumannii* is a recognized cause of healthcare-associated illness, including pneumonia, bacteremia, and urinary tract infections (UTIs) (*1*–*3*). *Acinetobacter* isolates often demonstrate resistance to multiple classes of antimicrobial drugs, leading to treatment challenges. A Centers for Disease Control and Prevention (CDC) report, Antimicrobial Resistance Threats in the United States, 2013, highlighted multidrug-resistant *Acinetobacter* as a serious threat that causes ≈7,000 infections and ≈500 deaths in the United States each year (*4*).

Carbapenems are often used to treat multidrug-resistant bacterial infections, such as those caused by *Acinetobacter* spp. Nearly half of *Acinetobacter* strains isolated from persons with healthcare-associated infections reported to the CDC National Healthcare Safety Network in 2014 were carbapenem-nonsusceptible (*5*). Infections with carbapenem-resistant *A. baumannii* have been associated with death rates as high as 52% (*6*–*10*).

Preventing the transmission of resistant organisms, including carbapenem-resistant *A. baumannii*, is a major public health priority (*11*). To identify opportunities for prevention, the Emerging Infections Program (EIP) conducts population- and laboratory-based surveillance for carbapenem-nonsusceptible *A. baumannii* in 8 US metropolitan areas through its Multi-site Gram-negative Surveillance Initiative (MuGSI). Our objective was to describe the epidemiology and estimate the crude population-based incidence of carbapenem-nonsusceptible *A. baumannii* during the first 4 years of surveillance.

## Methods

### Surveillance Catchment Area

Surveillance was population-based and conducted in 3 metropolitan areas in 2012: Atlanta, Georgia (estimated population 3,991,607); Minneapolis and St. Paul, Minnesota (1,761,282); and Portland, Oregon (1,766,135). Four more metropolitan areas were added in 2013: Denver, Colorado (2,694,886); Baltimore, Maryland (1,934,018); Albuquerque, New Mexico (676,685); and Rochester, New York (749,600). One other site, Nashville, Tennessee (1,653,871), was added in 2014. The total population under surveillance in 2015 was ≈15.2 million, which included 31 counties in the identified metropolitan areas (*12*). The project comprised cases for which samples were collected for culture of carbapenem-nonsusceptible *A. baumannii* during January 1, 2012–December 31, 2015.

### Case Definition and Epidemiologic Classification

We defined a case as the first isolation of carbapenem-nonsusceptible *A. baumannii* complex in a 30-day period from a normally sterile body site or urine specimen of a surveillance catchment area resident. Carbapenem nonsusceptibility was based on antimicrobial drug susceptibility test results generated by the local clinical laboratory’s primary testing method and 2012 Clinical and Laboratory Standards Institute interpretive criteria for meropenem and imipenem nonsusceptibility (MIC >2 μg/mL) (*13*). For doripenem, nonsusceptibility was defined using the 2012 Food and Drug Administration’s breakpoint (MIC >1 μg/mL) (https://druginserts.com/lib/rx/meds/doribax/page/3/). Most clinical laboratories in the EIP catchment area used automated testing instruments for primary antimicrobial susceptibility testing (*14*). Respiratory cultures, although clinically important for carbapenem-nonsusceptible *A. baumannii*, were not included as part of this surveillance.

We considered a sample collected for initial culture to be hospital-collected if it was collected in a short-stay acute-care hospital inpatient setting. We considered a sample to be other healthcare–collected if it was collected in any of the following settings: long-term care facility ([LTCF]; i.e., nursing home, skilled nursing facility, inpatient hospice, or physical rehabilitation facility); long-term acute-care hospital (LTACH); dialysis center; outpatient care center (i.e., outpatient surgery center, urgent care, private doctor’s office/clinic); or the emergency department or observation units in an acute-care hospital.

### Case Identification and Data Collection

To identify cases, we actively collected reports of all carbapenem-nonsusceptible *A. baumannii* isolates from clinical laboratories serving the catchment areas. We reviewed the patient address that accompanied the isolate to determine whether the patient resided in the surveillance catchment area. We abstracted inpatient and outpatient medical records using a standardized case report form. Information collected was patient demographics, location of sample collection, healthcare exposures, types of infection diagnosed, underlying conditions, and patient outcomes. Death was determined at discharge if the sample had been collected from a hospital inpatient; 30 days after the sample collection date if the sample was collected in an outpatient dialysis center, LTCF, or LTACH; and on the date of visit if the sample was collected in an outpatient setting. We calculated a Charlson Comorbidity Index score on the basis of underlying conditions abstracted from the medical record (*15*,*16*). We collected additional data elements if the sample was urine: method of urine collection, colony count of organisms isolated from the urine sample, and signs and symptoms documented in the medical record during the 2 calendar days before through the 2 calendar days after sample collection. We distinguished UTIs from colonization on the basis of the following criteria: 1) urine sample positive for >10^5^ CFU/mL carbapenem-nonsusceptible *A. baumannii*; and 2) signs or symptoms consistent with UTI documented in the medical record during the 2 calendar days before through the 2 calendar days after sample collection. We further categorized UTIs as catheter-associated if a urinary catheter was in place in the 2 days before sample collection and if the case-defining sample was a catheter urine specimen for the same organism.

### Statistics

To compare incidence between sites and over time, we linked annual case counts reported by each EIP surveillance site with annual US Census population counts for the corresponding counties. We also stratified counts from both data sources and linked them by age, sex, and race to enable adjustment of potential confounding factors. We imputed cases with missing values for race in accordance with the distribution of known race among patients by age category. Incidence rates, calculated from case counts, were expressed as number of infections per 100,000 population, and precision was quantified using 95% CIs assuming a Poisson distribution (*17*). We compared stratified rates for each site to the combined EIP population using standardized incidence ratios, an indirect method of rate standardization appropriate for small event counts. The combined EIP population served as the standard population. We calculated exact Poisson confidence intervals around the standardized incidence ratios using a formula relating the χ^2^ and Poisson distributions (*18*) and calculated p values using Miettinen’s modification for Mid-P exact test for counts <100 and Byar’s approximation of the Poisson method for counts >100 (*19*). We calculated adjusted incidence rates by multiplying the site-specific standardized incidence ratios adjusted for age, sex, and race by the crude incidence rate of the standard population (*20*). We assessed change in incidence over time using incidence rate ratios (IRRs) obtained from a Poisson regression model adjusting for site, age, race, and sex with 2013 as the reference year. We limited analysis to sites contributing data annually during 2013–2015 (i.e., we excluded Tennessee data).

Analyses were conducted to describe patients’ healthcare exposures, outcomes, demographic information, and antimicrobial drug susceptibility information for cases and for unique patients. Patients with complete case report form data as of August 26, 2016, were included in analyses of healthcare exposures and demographic and clinical characteristics and outcomes, and all cases (some patients contributed multiple cases to the analysis) were included in the antimicrobial drug susceptibility analysis. We calculated p values for comparison of categorical variables using the χ^2^ test or the Fisher exact test when cell size was <5. Data management and analyses were conducted using SAS version 9.4 (SAS Institute Inc., Cary, NC, USA).

### Ethics Review

Human subjects advisors in CDC’s National Center for Emerging and Zoonotic Infectious Diseases determined the EIP’s MuGSI to be a nonresearch activity; therefore, CDC institutional review board review was not required. This study also underwent ethics review at each of the participating EIP sites and was either approved with waiver of informed consent or deemed a nonresearch activity.

## Results

### Incidence Rates, Standardized Infection Rate Ratios, and Trends

The overall crude incidence rate was 1.2 (95% CI 1.1–1.3)/100,000 persons for 2012–2015 (Table 1). Crude incidence rates varied by EIP site during the 4-year period; the highest rates occurred in Maryland. Standardized incidence ratios were significantly higher than expected for Maryland (p = 0.00) and Georgia (p = 0.001) and significantly lower than expected for Colorado (p = 0.000), Minnesota (p = 0.000), New Mexico (p = 0.000), New York (p = 0.000), Oregon (p = 0.000), and Tennessee (p = 0.041) (Table 1). When we accounted for age, sex, race, and site, among the 7 sites that participated during 2013–2015, the adjusted IRRs did not differ (0.83 [95% CI 0.67–1.03]; p = 0.09). In 2014, the adjusted IRR decreased 24% from that of 2013 (0.76 [95% CI 0.61–0.95]; p = 0.02). During the surveillance period, 1 facility accounted for much of the decrease from 2013 to 2014, consistent with resolution of an outbreak. When we removed this facility from this temporal analysis, the change from 2013 to 2014 was no longer significant (adjusted IRR 0.82 [95% CI 0.66–1.03]; p = 0.09).

**Table 1:** Case counts and incidence of carbapenem-nonsusceptiable *Acinetobacter baumannii* in Emerging Infections Program sites, United States, 2012–2015*

Area	No. cases, N = 621					Crude annual incidence rates‡ (95% CI)				aSIR§ (95% CI)	aIR§ for all years
2012†	2013	2014	2015	2012†	2013	2014	2015
CO	ND	11	7	8		ND	0.4 (0.2–0.8)	0.3 (0.1–0.6)	0.3 (0.1–0.6)	0.4 (0.3–0.6)	0.4
GA	111	78	47	64		2.9 (2.4–3.5)	2.0 (1.6–2.5)	1.2 (0.9–1.5)	1.6 (1.2–2.1)	1.2 (1.1–1.4)	1.2
MD	ND	77	81	78		ND	4.0 (3.2–5.0)	4.2 (3.3–5.2)	4.0 (3.2–5.0)	2.5 (2.2–2.9)	3.0
MN	2	10	7	7		0.12 (0–0.4)	0.6 (0.3–1.1)	0.4 (0.2–0.8)	0.4 (0.2–0.8)	0.4 (0.2–0.5)	0.4
NM	ND	0	1	1		ND	0 (0–0.4)	0.2 (0–0.8)	0.2 (0–0.8)	0.1 (0–0.4)	0.1
NY	ND	4	1	3		ND	0.5 (0.2–1.4)	0.1 (0–0.7)	0.4 (0.1–1.2)	0.3 (0.1–0.6)	0.4
OR	0	4	0	0		0 (0–0.2)	0.2 (0.1–0.6)	0 (0–0.2)	0 (0–0.2)	0.1 (0–0.1)	0.1
TN	ND	ND	12	7		ND	ND	0.7 (0.4–1.3)	0.4 (0.2–0.9)	0.6 (0.4–1.0)	0.7
Total	113	184	156	168		1.6 (1.3–1.9)	1.4 (1.2–1.6)	1.0 (0.9–1.2)	1.1 (0.9–1.3)	ND	ND

*The study areas were Denver, CO; Atlanta, GA; Baltimore, MD; Minneapolis/Saint Paul, MN; Albuquerque, NM; Rochester, NY; Portland, OR; Nashville, TN. aIR, annual incidence rate; aSIR, adjusted standardized incidence ratio; ND, no data available. †Only 3 Emerging Infections Program sites participated in 2012. ‡Per 100,000 population. §Adjusted for age, race, and sex.

### Cases and Patients

A total of 621 carbapenem-nonsusceptible *A. baumannii* cases from 537 unique patients were reported during the study period. Most cases occurred in Georgia (300 [48.3%]), followed by Maryland (236 [38.0%]), Minnesota (26 [4.2%]), Colorado (26 [4.2%]), and Tennessee (19 [3.1%]). New York, Oregon, and New Mexico reported 8, 4, and 2 cases, respectively. Of the 537 patients, 119 (22.2%) had >2 carbapenem-nonsusceptible *A. baumannii* cultures; these repeat positives accounted for 203 of all cases during the 4-year surveillance period (range 2–8 cultures/patient).

Among the 513 patients with complete case report form data, 178 (34.7%) were female, and median age was 58.6 years (range 0–102 years). Information about underlying conditions was available for 512 patients. The median Charlson Comorbidity Index score was 2.9 (range 0–13). Sixteen (3.1%) patients had no identified underlying conditions at the time of sample collection. The following underlying conditions were reported in >25% of patients: neurologic problems (277 [54.0%]), decubitus or pressure ulcers (275 [53.6%]), diabetes (216 [42.1%]), and chronic pulmonary disease (139 [27.1%]).

Most of the 598 cases were based on isolation of carbapenem-nonsusceptible *A. baumannii* from urine (429 [71.7%]), followed by blood (157 [26.3%]). Other sterile sites from which carbapenem-nonsusceptible *A. baumannii* was isolated were bone (8 [1.3%]), joint/synovial fluid (2 [0.3%]), peritoneal fluid (2 [0.3%]), pleural fluid (1 [0.2%]), and other normally sterile sites (3 [0.5%]). For 4 cases, both blood and urine samples were collected on same date, and both grew carbapenem-nonsusceptible *A. baumannii*.

For most (503 [84.1%]) cases, at least 1 type of infection was associated with the carbapenem-nonsusceptible *A. baumannii* from the sample collected. Among those, UTI was the most common infection type reported (328 [65.2%]), followed by bacteremia (158 [31.4%]), septic shock (55 [10.9%]), and pneumonia (33 [6.6%]). For most (403 [80.1%]) cases, only 1 infection type was reported; 100 (19.9%) had >2 types of infection.

Of the infections in the 429 cases for which carbapenem-nonsusceptible *A. baumannii* was isolated from urine, 115 (26.8%) met criteria for a UTI based on our definition. Of these, fever was the only symptom reported in 76 (66%) cases. A provider documented UTI in the medical record for 328 (76.5%) cases; of these, only 96 (29.3%) met the criteria for a UTI based on our definition.

Of the 598 cases, 228 (38.1%) were identified from cultures of hospital-collected samples. Of these, 85 (14.2%) samples were collected in the intensive care unit (ICU), and 148 (24.8%) were collected >3 days after hospital admission. The median time between admission and sample collection was 7 days (range 0–341 days). The remaining 370 (61.9%) samples were collected outside of the acute-care hospital setting (other healthcare–collected): 210 (35.1%) in the emergency department of an acute-care hospital, 101 (16.9%) in an LTCF, 31 (5.2%) in an LTACH, and 28 (4.7%) in an outpatient setting (e.g., private doctors’ offices or clinics). Of the 210 cases with samples collected in an emergency department, 177 (84.2%) were subsequently admitted to the acute-care hospital.

### Previous Healthcare Exposure

For nearly all cases (590 [98.7%]), healthcare exposure in the year before sample collection or an indwelling device around the time of sample collection was documented (Table 2). Admission to a short-stay acute-care hospital (469 [78.4%] patients) or LTCF (360 [60.2%]) were the most frequent healthcare exposures documented. Additionally, 14.0% of cases occurred in patients admitted to an LTACH. Among the 506 (84.6%) cases for which an indwelling device was documented in the 2 days before specimen collection, a urinary catheter (399 [66.7%]) was the most common device. For 8 (<1%) cases, healthcare exposure during the previous year was not identified; of these, 2 case-patients traveled internationally in the 2 months be-fore sample collection.

**Table 2:** Previous healthcare exposures among 598 carbapenem-nonsusceptible *Acinetobacter baumannii* cases‡ in Emerging Infections Program sites, United States, 2012–2015*

Healthcare exposure	No. (%)
Healthcare facility exposure in the year before sample collection†	528 (88.3)
Previous acute care hospitalization	469 (78.4)
Residence in long-term care facility	360 (60.2)
Inpatient or outpatient surgery	199 (33.3)
Admission to long-term acute care hospital‡	73 (14.0)
Current hemodialysis treatment	66 (11.0)
Any indwelling device in place in the 2 calendar days before sample collection	506 (84.6)
Urinary catheter	399 (66.7)
Central venous catheter	222 (37.1)
Other§	269 (45.0)
No healthcare exposure	8 (1.3)

*The study areas were Denver, CO; Atlanta, GA; Baltimore, MD; Minneapolis/Saint Paul, MN; Albuquerque, NM; Rochester, NY; Portland, OR; Nashville, TN. †Sum of subcategory percentages >100 due to patients with multiple healthcare exposures in year before case. ‡2013–2015 cases only. §Other indwelling devices: endotracheal or nasotracheal tube, tracheostomy, gastrostomy tube, nephrostomy tube, nasogastric tube.

### Outcomes of Cases

For 449 (75.1%) cases, patients were hospitalized at the time of or within 30 days after sample collection (Table 3). Of these cases, 168 (37.4%) patients were admitted to the ICU on the day of or within 7 days after sample collection. Death was assessed at different time points depending on where the patient was treated. The overall death rate of 17.9% (106/594 cases) was significantly higher for cases for which carbapenem-nonsusceptible *A. baumannii* was isolated from a sterile site than for those for which carbapenem-nonsusceptible *A. baumannii* was isolated only from urine (41.3% vs 8.3%; p<0.0001). Among case-patients who died, carbapenem-nonsusceptible *A. baumannii* was isolated within 7 days of death for 61.3% (65/106).

**Table 3:** Outcomes for 598 *Acinetobacter baumannii* cases in Emerging Infections Program sites, United States, 2012–2015*

Outcome	No. (%)
Hospitalized at, or within 30 d after, date of specimen collection, n = 598	449 (75.1)
Admission to intensive care unit on day of or within 7 d after sample collection, n = 449	168 (37.4)
Discharge location after acute care hospitalization among patients who survived, n = 356†	
Long-term care facility	187 (52.5)
Private residence	131 (36.8)
Long-term acute care hospital	34 (9.6)
Other	1 (0.3)
Died,‡ n = 594	106 (17.9)
Among cases with a sterile site culture, n = 172§	71 (41.3)
Among cases with a positive urine culture, n = 422§	35 (8.3)

*The study areas were Denver, CO; Atlanta, GA; Baltimore, MD; Minneapolis/Saint Paul, MN, Albuquerque, NM; Rochester, NY; Portland, OR; Nashville, TN. †Three case-patients were discharged to unknown locations. ‡Death was determined at discharge for hospital inpatients; 30 d after sample collection for case-patients identified in outpatient dialysis, long-term care, and long-term acute care hospitals; and at evaluation for outpatients. For 4 case-patients, outcome was unknown. The 1 patient who had a blood sample and a urine sample was counted in the “sterile site culture” category. §Significant difference in death by specimen source (p<0.0001).

### Antimicrobial Drug Susceptibilities

Antimicrobial drug susceptibility information from local clinical laboratories was available for all 621 cases (Table 4). Most isolates were susceptible to at least 1 aminoglycoside (72.9%). Isolates from urine samples were significantly more likely than those from sterile site samples to be susceptible to fluoroquinolones (4.6% vs. 0.6%; p = 0.02); susceptibilities based on specimen source did not differ significantly for other antimicrobial drugs.

**Table 4:** Antimicrobial susceptibility of 621 carbapenem-nonsusceptible *Acinetobacter baumannii* isolates reported by local clinical laboratories in Emerging Infections Program sites, United States, 2012–2015*

Antimicrobial agent	No. susceptible isolates/no. isolates tested (%)			p value†
	Total (%)	Sterile site cultures, n = 173	Urine cultures, n = 425	
Any aminoglycoside‡	421/577 (72.9)	123/166 (74.1)	298/411 (72.5)	0.70
Tobramycin	308/541 (56.9)	92/158 (58.2)	216/383 (56.4)	0.70
Amikacin	254/416 (61.1)	71/121 (58.7)	183/295 (62.0)	0.52
Gentamicin	175/571 (30.7)	49/163 (30.1)	126/408 (30.9)	0.85
Any fluoroquinolone‡	20/575 (3.5)	1/164 (0.6)	19/411 (4.6)	0.02
Levofloxacin	15/432 (3.5)	1/120 (0.8)	14/312 (4.5)	0.08
Ciprofloxacin	10/522 (1.9)	1/145 (0.7)	9/377 (2.4)	0.30
Any extended-spectrum β-lactam‡	114/577 (19.8)	27/165 (16.4)	87/412 (21.1)	0.20
Ceftazidime	72/447 (16.1)	17/129 (13.2)	55/318 (17.3)	0.28
Cefepime	68/562 (12.1)	18/161 (11.2)	50/401 (12.5)	0.67
Piperacillin/tazobactam	4/113 (3.5)	1/37 (2.7)	3/76 (1.9)	>0.99
Other				
Ampicillin/sulbactam	180/498 (36.1)	47/146 (32.2)	133/352 (37.8)	0.24
Colistin	114/122 (93.4)	36/39 (92.3)	78/83 (94.0)	0.73
Trimethoprim/sulfamethoxazole	83/483 (17.2)	26/142 (18.3)	57/341 (16.7)	0.67
Tigecycline	74/120 (61.7)	27/40 (67.5)	47/80 (58.8)	0.35

*The study areas were Denver, CO; Atlanta, GA; Baltimore, MD; Minneapolis/Saint Paul, MN, Albuquerque, NM; Rochester, NY; Portland, OR; Nashville, TN. †The χ^2^ test or Fisher exact test was used to test the null hypothesis “carbapenem-nonsusceptible *A. baumannii* isolates antimicrobial susceptibility did not differ for sterile site vs. urine cultures.” Fisher exact test was used when >1 cell size was <5. ‡Includes the antimicrobial drugs listed below.

## Discussion

Data from population-based surveillance covering 8 geographically diverse metropolitan areas in the United States show that the overall incidence of carbapenem-nonsusceptible *A. baumannii* infection during 2012–2015 was low (1.2 cases/100,000 persons). Cases occurred almost exclusively in patients who stayed overnight in healthcare facilities or had indwelling devices. The crude mortality rate was 17.9%, approximately double that for patients with carbapenem-resistant *Enterobacteriaceae* (CRE) from the same catchment areas (*21*). For most cases, samples for culture were collected outside of short-stay acute-care hospitals, indicating that efforts to prevent transmission should include a variety of healthcare settings. These unique data, which include clinical data and isolate reports from a variety of healthcare settings and patients, highlight potential opportunities to reduce transmission.

The incidence rates for carbapenem-nonsusceptible *A. baumannii* are lower than those reported from the identical EIP catchment areas for CRE (2.93 cases/100,000 population) (*21*) and substantially lower than rates reported from EIP for invasive methicillin-resistant *Staphylococcus aureus* (25.1/100,000) (*22*) and for *Clostridium difficile* (141.77/100,000) infections (*23*). The reasons for the lower incidence of carbapenem-nonsusceptible *A. baumannii* in this population than for other healthcare-associated pathogens are not clear but might be related to the low virulence of carbapenem-nonsusceptible *A. baumannii* (*2*) and the lack of dominant, well-adapted clones capable of spreading easily from person to person or within healthcare environments in our specific surveillance areas (*1*,*2*,*24*). However, because this surveillance is population-based, we were unable to measure the incidence of carbapenem-nonsusceptible *A. baumannii* within individual healthcare facilities, where it could be substantial.

Nearly all incident carbapenem-nonsusceptible *A. baumannii* cases were healthcare-associated; the most common exposures were admission to a short-stay acute-care hospital or residence in an LTCF during the previous year or the presence of an indwelling device. Similarly, Zeana et al. found multidrug-resistant phenotypes only among the hospital strains of *A. baumannii* collected from 2 US hospitals and from the community (*25*). Our findings support current recommendations to focus on preventing *A. baumannii* transmission in long-term care and acute-care hospital settings (*26*). In addition, the large proportion of patients transferred to LTCFs (60.2%) highlights the critical need for reporting patient multidrug-resistant organism status at interfacility transfer to ensure no gaps exist in the application of appropriate precautions. We observed substantial heterogeneity in adjusted incidence rates among EIP sites: a 20-fold difference between the site with the highest incidence (Maryland, 2.29 cases/100,000 persons) and the site with the lowest incidence (Oregon, 0.07/100,000). Similar geographic heterogeneity has been described with CRE (*21*) and might reflect several factors, including the underlying resistance mechanisms present or circulating among organisms in a specific location, length of time the organisms have been present in the region, and the implementation of infection control interventions to control spread.

Yearly adjusted incidence rates did not change significantly during 2013–2015 in the EIP surveillance catchment area. Although not always concordant with changes in incidence rates, the percentage of *Acinetobacter* spp. resistant to a carbapenem from healthcare-associated infections reported to the National Healthcare Safety Network decreased slightly from 2011 to 2014; in 2014, the percentage of *Acinetobacter* spp. nonsusceptible to a carbapenem was 50%, compared with 58% in 2011 (*5*,*27*). By contrast, before 2012, multiple US reports documented increases in resistant *Acinetobacter* (*28*–*30*). In a small study of clinical isolates conducted in Detroit during 2003–2008, the total number of patients with *A. baumannii* increased from 1.7/1,000 patient days in 2003 to 3.7/1,000 patient days in 2008; among these same patients the percentage of *Acinetobacter* isolates that were susceptible to imipenem decreased from 99% in 2003 to 42% in 2008 (*29*). In another US study of susceptibility results from hospital clinical microbiology laboratories contributing data to the Eurofins laboratory testing network across the United States, the percentage of *A. baumannii* isolates that were resistant to carbapenems increased from 21% in 2003–2005 to 48% in 2009–2012 (*30*). The relatively small number of cases and relatively short interval in our evaluation preclude us from identifying a clear trend in disease; additional years of surveillance data are needed to clarify these trends and the factors contributing to resistance and incidence differences across geographic regions.

Antimicrobial drug susceptibility testing performed at local laboratories demonstrated high levels of resistance to other antimicrobial drugs in addition to carbapenems. Most isolates were also nonsusceptible to cephalosporins, fluoroquinolones, trimethoprim/sulfamethoxazole, ampicillin/sulbactam, and piperacillin/tazobactam. Most remained susceptible to at least 1 aminoglycoside and, for the subset for which a result was available, to colistin and tigecycline. The 3 drug classes to which most isolates were susceptible can be associated with substantial toxicities or treatment failure (*29*) and are generally considered second-line agents for treatment. Although we did not collect data on carbapenem-nonsusceptible *A. baumannii* infection treatment and were unable to determine the proportion of deaths attributable to *Acinetobacter* infection, the limited availability of drugs to which carbapenem-nonsusceptible *A. baumannii* isolates were susceptible could have contributed to the overall death rate of 41% for cases for which carbapenem-nonsusceptible *A. baumannii* was isolated from a sterile site.

Our findings are subject to several limitations. First, because not all local clinical laboratories serving the catchment area participated during the entire period, these results underestimate the true incidence of carbapenem-nonsusceptible *A. baumannii*, particularly among specific populations, such as dialysis patients and LTCF residents. Second, although *Acinetobacter* can be isolated from sputum and other nonsterile sites, these sources were not included in the surveillance, which resulted in an underestimation of the total number of cases. Third, we did not collect carbapenem-nonsusceptible *A. baumannii* isolates and therefore were unable to describe resistance mechanisms. A better understanding of these mechanisms could inform prevention and control strategies. Going forward, isolate collection through CDC’s Antimicrobial Resistance Laboratory Network will help to define *Acinetobacter* resistance mechanisms in the United States. Fourth, although 15 million persons live in the areas under surveillance, the data demonstrate considerable geographic heterogeneity; therefore the results of this analysis might not be generalizable to all areas of the United States. Fifth, use of the population of the catchment area is an imperfect denominator to represent the burden of disease attributable to a pathogen largely concentrated within selected healthcare facilities. Sixth, data were retrospectively abstracted from medical records, and the quality and completeness of such records can vary among healthcare systems and facility types, resulting in underreporting of some data elements. Finally, our incidence case definition was based on a 30-day period; extending the interval between incident cases or excluding recurrent cases would have resulted in a lower incidence rate.

In summary, we present population-based carbapenem-nonsusceptible *A. baumannii* incidence rates in the United States and provide additional information about the epidemiology of carbapenem-nonsusceptible *A. baumannii*. These data, along with data from the National Healthcare Safety Network, provide early evidence that carbapenem resistance among *A. baumannii* isolates might have plateaued, although additional years of surveillance in both systems are needed to confirm this observation. Despite a currently low population-based incidence, the medical complexity of carbapenem-nonsusceptible *A. baumannii *patients, along with treatment challenges posed by high levels of resistance to noncarbapenem antimicrobial drugs and high death rates, highlight the need for additional work in healthcare settings to contain carbapenem-nonsusceptible *A. baumannii* spread.
